# Expression and prognostic significance of MYL9 in esophageal squamous cell carcinoma

**DOI:** 10.1371/journal.pone.0175280

**Published:** 2017-04-07

**Authors:** Jian-Hua Wang, Lan Zhang, Shu-Ting Huang, Jing Xu, Yun Zhou, Xing-Juan Yu, Rong-Zhen Luo, Zhe-Sheng Wen, Wei-Hua Jia, Min Zheng

**Affiliations:** 1 Department of Chest, Guangdong Second Provincial General Hospital, Guangzhou, Guangdong, P. R. China; 2 Department of Gynecology, Sun Yat-Sen University Cancer Center, Guangzhou, Guangdong, P. R. China; 3 State Key Laboratory of Oncology in South China, Guangzhou, Guangdong, P. R. China; 4 Collaborative Innovation Center for Cancer Medicine, Guangdong, Guangzhou, P. R. China; 5 Department of Radiation Oncology, The Third Affiliated Hospital of Kunming Medical University, Yunnan Tumor Hospital, Kunming, Yunnan, PR China; 6 Department of Pathology, Sun Yat-Sen University Cancer Center, Guangzhou, Guangdong, P. R. China; 7 Department of Chest, Sun Yat-Sen University Cancer Center, Guangzhou, Guangdong, P. R. China; University of North Carolina at Chapel Hill School of Medicine, UNITED STATES

## Abstract

**Objective:**

Myosin light chain 9 (MYL9) is necessary for cytoskeletal dynamics and experimental metastasis, but its expression in esophageal squamous cell carcinoma (ESCC) has not been addressed. We investigated the expression pattern and clinical significance of MYL9 in patients with ESCC.

**Methods:**

We examined MYL9 expression using quantitative real-time PCR and western blotting in NE1 immortalized esophageal epithelial cells, ESCC cell lines, and paired ESCC tissues. MYL9 protein in 136 primary ESCC tissues and other types of solid tumor was detected using immunohistochemistry. The association between MYL9 expression and clinical parameters and survival was evaluated by statistical analysis.

**Results:**

MYL9 was significantly upregulated in the ESCC cell lines as compared with NE1 cells. In the paired ESCC samples, MYL9 mRNA and protein expression was not significantly different between lesion tissues and the matched adjacent noncancerous tissues. In ESCC tissue, both intratumoral and peritumoral stroma were positive for MYL9. In the 136 ESCC samples, high MYL9 expression in the tumor cells significantly correlated with histological differentiation (*p* = 0.028), recurrence (*p* = 0.01), and vital status (*p* < 0.01). Patients with high MYL9 expression in the tumor cells had poorer overall survival (OS) and recurrence-free survival. Multivariate analysis revealed that high MYL9 expression in tumor cells was an independent and significant risk factor affecting OS after curative treatment (hazard ratio = 2.254, 95% confidence interval = 1.347–3.771, *p* = 0.002).

**Conclusions:**

MYL9 expression might be a promising prognostic marker and therapeutic target in ESCC.

## Introduction

Esophageal cancer is a major cancer burden; its annual incidence rate is approximately 481,600 and 259,200 cases worldwide and in China, respectively [1, 2]. Histologically, up to 90% of esophageal cancer cases worldwide are esophageal squamous cell carcinoma (ESCC). Despite the progress in adjuvant chemoradiation, targeted therapy, and surgical treatment, the 5-year survival rate remains less than 25%, largely due to late diagnosis and the propensity for metastasis [3]. Accumulating evidence shows that a variety of biological abnormalities, including altered gene expression, gene mutations, aberrant signaling pathways, and genetic alterations, contribute to ESCC development and progression. However, reliable and reproducible prognostic markers identifying patients at high risk of ESCC recurrence after surgery have not been established. A better understanding to the biology of ESCC recurrence is needed to improve patient care.

Myosins, actin-dependent molecular motors that utilize the energy of adenosine triphosphate hydrolysis to generate force, play key roles in regulating tumor progression and metastasis[4]. Myosin superfamily members can enhance or suppress tumor progression [5, 6]. The first myosin to be studied biochemically was myosin II[7]. Myosin II is a hexameric molecule consisting of two heavy chains and two sets of paired light chains: the essential light chain and the regulatory light chain. Myosin II activity is mainly regulated via post-translational phosphorylation of myosin light chain 9 (MYL9, also known as MLC2, MRLC1, or MLC-2C) by the opposing activities of MLC kinases and a MLC phosphatase [8]. Recently, MYL9 was shown to be necessary for cytoskeletal dynamics and experimental metastasis [9]. For example, MYL9 was regulated by the myocardin-related transcription factor–serum response factor (MRTF-SRF) pathway and was required for tumor cell, megakaryocyte, and tip cell migration *in vivo*[9–12]; MYL9 overexpression resulted in invasion-promoting functions of cancer-associated fibroblasts [13], and the introduction of exogenous early growth response 1 (EGR1) augmented the metastatic potential of non-metastatic PR9692-E9 cells by activating MYL9[14], while microRNA-10b overexpression or direct knockdown of syndecan-1 increased cancer cell migration and Matrigel invasiveness accompanied by MYL9 upregulation[15].

Despite much evidence supporting the promoter role of MYL9 in tumor invasion and metastasis [9, 10, 14–16], it has been demonstrated that its clinical significance in human tissues differs by tumor type. In bladder cancer, colon cancer, non–small cell lung cancer, and prostate cancer, total MYL9 expression was downregulated in tumor tissues compared with normal tissues[17–20]. Low MYL9 expression correlated with poor survival in colon and prostate cancer, suggesting that MYL9 is a favorable prognostic marker [18, 20]. On the other hand, high MYL9 expression was significantly associated with late tumor-node-metastasis (TNM) stage and lymphatic metastasis in non–small cell lung cancer[19]. Nevertheless, studies on the association between MYL9 expression and cancer are rare, and there has been no published report on the characteristics of MYL9 expression and its clinical significance in ESCC.

Therefore, we aimed to explore the expression pattern of MYL9 in ESCC cell lines and ESCC specimens. Further, we investigated the relationship between MYL9 and the clinicopathological parameters of ESCC and analyzed its prognostic value based on outcome data.

## Methods

### Ethics statement

The research ethics committee of the Sun Yat-sen University Cancer Center (Guangzhou, China) provided ethical approval for this study, and all patients provided written informed consent. All specimens were handled and stored anonymously according to ethical and legal standards.

### Patients and specimens

Tumor samples were obtained from patients with pathologically confirmed ESCC (*n* = 136), cervical (*n* = 20), ovarian (*n* = 20), colorectal (*n* = 40), and hepatocellular carcinoma (*n* = 10) at the Sun Yat-sen University Cancer Center in 2002–2009. No patient had received anti-tumor therapy before sampling. The clinicopathological parameters of 136 patients with ESCC were obtained from medical records and pathology reports. ESCC specimens were staged in accordance with American Joint Cancer Committee/Union International Contre le Cancer (UICC/AJCC) classification guidelines. The grading and histopathology subtyping of ESCC specimens was based on World Health Organization criteria. Patient consent was obtained prior to the use of the clinical materials for research purposes. The Sun Yat-sen University Cancer Center Institutional Review Board approved the study and it was conducted in accordance with the Declaration of Helsinki. Patients attended follow-up visits regularly. Data were censored at the last follow-up for patients without recurrence or death. Overall survival (OS) was defined as the interval between surgery and death or the last observation. Recurrence-free survival (RFS) was defined as the date of surgery to recurrence, the last follow-up for patients without recurrence, or death if no recurrence was observed.

### Cell lines

The ESCC cell lines KYSE30, KYSE140, KYSE180, KYSE410, KYSE510, and KYSE520 were obtained from Deutsche Sammlung von Mikroorganismen und Zellkulturen, the German Resource Center for Biological Material[21]. TE1, HKESC1, EC18, and EC109 cells and the NE1 immortalized esophageal epithelial cell line were kept in the State Key Laboratory of Oncology in South China, Sun Yat-sen University Cancer Center. The cell lines were cultured in Dulbecco’s modified Eagle’s medium (DMEM; Life Technologies, Carlsbad, CA, USA) supplemented with 10% fetal bovine serum (FBS; Life Technologies) in 5% CO_2_ at 37°C.

### RNA isolation and quantitative PCR (qPCR)

Total RNA was isolated from ESCC cell lines using an EasyPure RNA Kit (TransGen Biotech, Beijing, China) according to the manufacturer’s instructions. RNA (2 μg) was reverse-transcribed using MMLV reverse transcriptase reagents (Promega, Madison, WI, USA). For the qPCR assay, complementary DNA was PCR-amplified using a GoTaq qPCR Master Mix (Promega) in a LightCycler 480 II PCR system (Roche Diagnostics, Rotkreuz, Switzerland). Glyceraldehyde-3-phosphate dehydrogenase (GAPDH) was used as the internal control. The *MYL9* sense and anti-sense primers were 5′-CACCAGAAGCCAAGATGTCC-3′ and 5′-TTGAAAGCCTCCTTAAACTCC-3′, respectively. The *GAPDH* sense and anti-sense primers were 5′-GAAGGTGAAGGTCGGAGT-3′ and 5′-GAAGATGGTGATGGGATTTC-3′, respectively. Relative gene expression was presented as the comparative threshold cycle (2^-ΔΔCt^) values and was representative of at least three independent experiments.

### Western blotting

Protein was extracted using a protein extraction kit (KGP250-2100, KeyGen Biotech, Nanjing, China) according to the manufacturer’s instructions. Protein samples were treated with Dual Color Protein Loading Buffer (Thermo Fisher Scientific, Waltham, MA, USA) containing reducing agent at 100°C for 5 minutes, resolved on 10% Tris–HCl polyacrylamide gels, and transferred to a polyvinylidene fluoride membrane. Overnight incubation (4°C) with primary antibodies against MYL9 (1:1000; ab191393, Abcam, Cambridge, UK) and GAPDH (Abgent, San Diego, CA, USA) was followed by incubation (37°C) with horseradish peroxidase (HRP)-conjugated anti-rabbit (1:1000; Novus Biological, Littleton, CO, USA) or anti-mouse antibody (1:1000; Novus Biological) and Immobilon Western Chemiluminescent HRP Substrate (Millipore, Billerica, MA, USA) and a Tanon 5200 Luminescent Imaging Workstation (Tanon, Shanghai, China).

### Immunohistochemistry (IHC)

Formalin-fixed, paraffin-embedded samples were cut into 5-μm sections and processed for IHC. Tissue sections prepared for antigen retrieval by microwave treatment in EDTA buffer (pH 9.0) were incubated with primary antibodies against MYL9 (1:1000; ab191393, Abcam) and α-smooth muscle actin (SMA) (1:500; Zhongshan Bio-Tech, Zhongshan, China). Immunostaining was performed using the EnVision System with diaminobenzidine (Dako Cytomation, Glostrup, Denmark). Images were viewed and assessed using a microscope (Ecilipse 80i, Nikon, Tokyo, Japan). MYL9 expression in tumor cells was measured via the H-score method. The H-score was obtained as follows: (3 × percentage of strongly stained tumor cells) + (2 × percentage of moderately stained tumor cells) + (percentage of weakly stained tumor cells), yielding an H-score of 0–300. The cut-off value for high and low expression was 40 based on a measure of heterogeneity with log–rank statistical analysis with respect to OS. MYL9 staining on the stroma was not taken into account. Staining with isotype antibody was used as the negative control.

### Statistical analysis

Pearson’s χ^2^ test or Fisher’s exact test was used to examine the relationship between MYL9 expression and clinicopathological parameters as appropriate. Survival curves were calculated using the Kaplan–Meier method and analyzed using the log–rank test. Prognostic factors were examined by univariate and multivariate analyses using the Cox proportional hazards model. A *p*-value of less than 0.05 was considered statistically significant. All statistical analyses were performed using SPSS version 18.0 (SPSS, Chicago, IL, USA).

## Results

### MYL9 expression in ESCC cell lines and tissues

**Fig 1:**
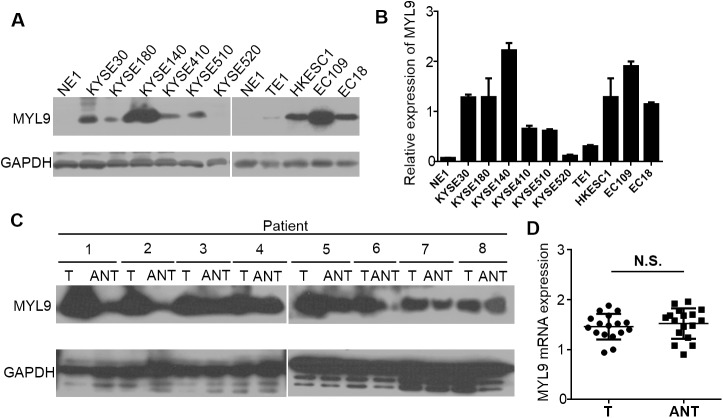
Western blotting and qPCR analyses of MYL9 expression in ESCC cell lines and tissues. (A) MYL9 protein and (B) mRNA expression levels in NE1 and ESCC cell lines. Expression levels were normalized against GAPDH. Error bars represent the standard deviation of the mean (SD), which was calculated from three parallel experiments. (C) Representative western blots of MYL9 protein expression in eight matched pairs of ESCC tissues (T) and adjacent noncancerous tissues (ANT). GAPDH was used as the loading control. (D) Statistical analyses of *MYL9* mRNA expression in matched paired T and ANT. N.S., not significant.

MYL9 expression levels in the nine ESCC cell lines (KYSE30, KYSE180, KYSE140, KYSE410, KYSE510, KYSE520, HKESC1, EC18, EC109) were assessed and compared with NE1 cells by western blotting and qPCR. MYL9 expression was barely detectable in the NE1 cells, whereas all ESCC cell lines had significantly higher MYL9 mRNA and protein levels (Fig 1A and 1B), demonstrating that MYL9 is upregulated in ESCC cell lines.

To examine whether MYL9 is also highly expressed in human ESCC clinical samples, we performed western blotting on eight ESCC tumor samples that were matched with adjacent noncancerous tissue samples (ANT). MYL9 expression in the tumor samples was higher than that in the paired ANT, but not very obviously so in some paired samples (Fig 1C). However, initial analysis of 17 paired ESCC samples from NCBI/GEO/GES20347 showed that *MYL9* mRNA expression was not significantly different between the ESCC tumor tissues and the matched ANT (*p* = 0.5310) (Fig 1D).

### IHC staining of MYL9 in ESCC tissues

**Fig 2:**
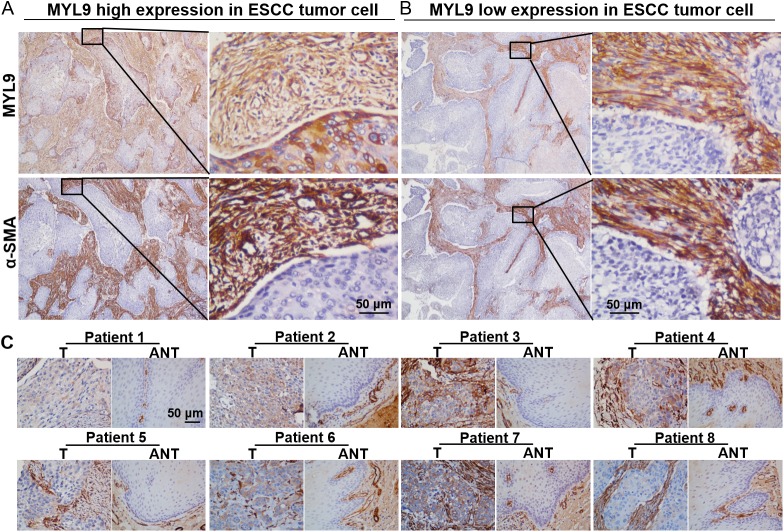
IHC characteristics of MYL9 in ESCC specimens. (A, B) Stromal cells in tumor tissues were positive for MYL9. Anti–α-SMA staining was used to distinguish stromal regions. (A) Representative staining of high MYL9 expression in tumor cell cytoplasm (left, ×40 magnification; right, ×400 magnification). (B) Representative staining of low MYL9 expression in tumor cell cytoplasm (left, ×40 magnification; right, ×400 magnification). Scale bar = 50 μm. (C) IHC assay of MYL9 protein expression in eight paired ESCC tissues. Scale bar = 50 μm.

As the western blotting and qPCR analyses did not reflect MYL9-positive cells *in situ*, we performed immunohistochemical staining to investigate the expression pattern of MYL9 in a retrospective cohort of 136 ESCC cases. In all samples, the stromal cells, distinguished by anti–α-SMA staining, were MYL9-positive in both the intratumoral (Fig 2A and 2B) and peritumoral regions (S1 Fig). Epithelial cells in the adjacent noncancerous regions were all stained negatively or minimally for MYL9 (Fig 2C). However, MYL9 expression levels in cancer cell cytoplasm varied widely among ESCC specimens (Fig 2C). MYL9 expression was often higher in tumor cells at the edge of the tumor tissue (S2 Fig). Therefore, we focused on aberrant MYL9 expression in tumor cells to investigate its clinical value.

We also explored the MYL9 expression patterns in cervical (*n* = 20), ovarian (*n* = 20), colorectal (*n* = 40), and hepatocellular (*n* = 10) carcinoma samples. MYL9 immunostaining was observed mainly in the cytoplasm of stromal cells and some tumor cells (S3 Fig), which was consistent with the expression pattern in ESCC tissues.

### Association between MYL9 expression and ESCC clinicopathological characteristics

Table 1 summarizes the clinicopathological features of the 136 patients with ESCC. The median follow-up was 43.9 months (range, 1.6–130.9 months). During the follow-up period, 69 patients (50.1%) died and 79 patients (58.1%) were diagnosed with tumor recurrence. The median OS and RFS was 44 and 33 months, respectively. Based on MYL9 expression levels in the tumor cell cytoplasm, the patients were divided into two groups: high MYL9 expression (high expression in tumor cells; Fig 2A) and low MYL9 expression (low expression in tumor cells; Fig 2B). MYL9 protein expression was high in 71 of 136 (41.2%) samples.

**Table 1:** Patient clinicopathological characteristics and MYL9 expression in ESCC.

	Cases (%)
***Sex***	
Male	94(69.1)
Female	42(30.9)
***Age (years)***	
<60	72(52.9)
≥60	64(47.1)
***Clinical stage***	
I	3(2.2)
IIA	53(39.0)
IIB	19(14.0)
III	61(44.8)
IV	0(0)
***T classification***	
T1	5(3.7)
T2	28(20.6)
T3	96(70.6)
T4	7(5.1)
***N classification***	
N0	67(49.3)
N1	68(50)
N2	1(0.7)
***M classification***	
M0	130(100)
M1	0(0)
***Differentiation***	
Well	29(21.3)
Moderate	70(51.5)
Poor	37(27.2)
***Recurrence***	
No	57(41.9)
Yes	79(58.1)
***Vital status (at follow-up)***	
Alive	67(49.3)
Death (tumor-related)	67(49.3)
Death (tumor-unrelated)	2(1.4)
***MYL9 expression***	
Low	65(47.8)
High	71(52.2)
***Location***	
Upper	10(7.4)
Middle	89(65.4)
Lower	37(27.2)
***Therapy***	
Surgery only	129(94.9)
Surgery + CT or RT or CRT	7(7.6)
***Completeness of surgical resection***	
Yes	136(100)
No	0(0)

Abbreviations: CT, chemotherapy; RT, radiotherapy; CRT, combination of CT and RT (chemoradiotherapy).

**Fig 3:**
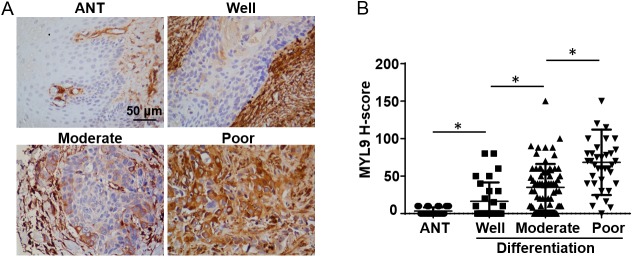
Association between MYL9 expression levels and tumor differentiation. Representative staining of MYL9 expression in adjacent noncancerous tissue (ANT) and in well-, moderately, and poorly differentiated tumors. Scale bar = 50 μm. (B) The average MYL9 staining score was increased from well-differentiated to poorly differentiated tumors, and was significantly higher than that in ANT regions. IHC H-scores are expressed as the mean ± SD (bars); *p < 0.05.

We analyzed the relationship between MYL9 expression levels in tumor cells and the clinicopathological characteristics. Table 2 shows that MYL9 protein expression and patient sex, age, clinical stage, T classification, N classification, and tumor location were not significantly correlated. However, MYL9 expression was significantly associated with histological differentiation (*p* = 0.028), recurrence (*p* = 0.01), and vital status (*p* < 0.01). Patients with poorly differentiated tumors tended to have higher MYL9 expression (Fig 3A and 3B).

**Table 2:** Correlation between MYL9 expression and clinicopathological characteristics of ESCC.

Characteristic		MYL9 expression		*p*
		Low	High	
		No.cases (%)	No.cases (%)	
***Gender***	Male	47(72.3)	47(66.2)	0.463
	Female	18(27.7)	24(33.8)	
***Age (years)***	<60	36(55.4)	36(50.7)	0.61
	≥60	29(44.6)	35(49.3)	
***Clinical stage***	I-II	37(56.9)	38(53.5)	0.732
	III	28(43.1)	33(46.5)	
***T classification***	T1-T2	15(23.1)	18(25.4)	0.842
	T3-T4	50(76.9)	53(74.6)	
***N classification***	N0	34(52.3)	33(46.5)	0.607
	N1-2	31(47.7)	38(53.5)	
***Differentiation***	Well or Moderate	53(81.5)	46(64.8)	**0.028**
	Poor	12(18.5)	25(35.2)	
***Location***	Upper	7(10.8)	3(4.2)	0.317
	Middle	42(64.6)	47(66.2)	
	Lower	16(24.6)	21(29.6)	
***Recurrence***	No	37(56.9)	20(28.2)	**0.01**
	Yes	28(43.1)	51(71.8)	
***Vital status***	Alive	44(67.7)	23(32.4)	<**0.01**
**(at follow-up)**	Death (tumor-related)	21(32.3)	46(64.8)	
	Death (tumor-unrelated)	0(0)	2(2.8)	
***Therapy***	Surgery only	62(95.4)	67(94.4)	0.549
	Surgery+CT or RT or CRT	3(4.6)	4(5.6)	

*p*-values were analyzed by χ^2^ test or Fisher’s exact test, as appropriate.

Abbreviations: CT, chemotherapy; RT, radiotherapy; CRT, combination of CT and RT (chemoradiotherapy).

### High MYL9 expression in tumor cells predicts poor prognosis

Univariate analysis of MYL9 status and conventional clinicopathological parameters for prognosis showed that high MYL9 expression, advanced clinical stage, and T and N classification were unfavorable predictors of OS in ESCC (Table 3). Positive MYL9 expression and advanced clinical stage were independent prognostic factors for ESCC (Table 3).

**Table 3:** Univariable and multivariable analyses of prognostic parameters in patients with ESCC.

Variable		HR (95% CI)	*p*
Univariable analysis			
***Clinical stage***	I-II/III	2.076(1.288–3.345)	**0.003**
***T classification***	T1-T2/T3-T4	1.948(1.043–3.639)	**0.036**
***N classification***	N0/N1-2	1.882(1.159–3.056)	**0.011**
***Differentiation***	Well or Moderate/Poor	1.550(0.938–2.564)	0.087
***Location***	Upper/Middle/Lower	1.314(0.854–2.022)	0.214
***Therapy***	Surgery only/Surgery+CT or RT or CRT	0.178(0.025–1.280)	0.086
***MYL9 expression***	Negative/Positive	2.307(1.379–3.858)	**0.001**
Multivariable analysis			
***Clinical stage***	I-II/III	2.307(1.379–3.858)	**0.004**
***MYL9 expression***	Negative/Positive	2.254(1.347–3.771)	**0.002**

Univariate analysis, Cox proportional hazards regression model.

Multivariate analysis, Cox proportional hazards regression model. Variables were adopted by univariate analysis.

Abbreviations: HR, hazard ratio; CI, confidence interval; CT, chemotherapy; RT, radiotherapy; CRT, combination of CT and RT (chemoradiotherapy).

**Fig 4:**
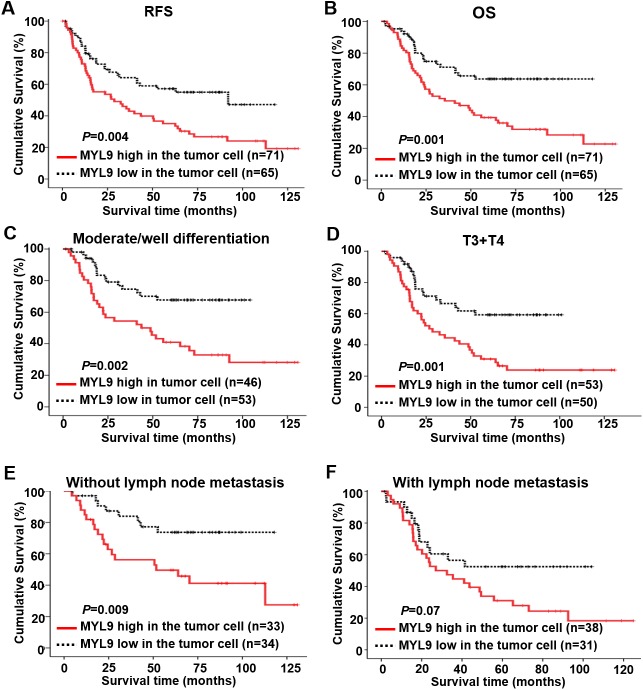
Kaplan–Meier curves of univariate analysis data (log–rank test). (A, B) The RFS (A) and (B) OS of patients with high versus low MYL9 expression. (C) The OS for moderately/well-differentiated patients with high versus low MYL9 expression. (D) The OS for patients with T3+T4 classification with high versus low MYL9 expression. (E, F) The OS for patients without lymph node metastasis (E) and with lymph node metastasis (F) with high versus low MYL9 expression.

Kaplan–Meier analysis and the log–rank test showed that patients with high MYL9 expression had shorter RFS (*p* = 0.004, Fig 4A) and worse OS (*p* = 0.001, Fig 4B) than patients with low MYL9 expression. The 5-year RFS and OS rates for the patients with high MYL9 expression were 50% and 64%, respectively, whereas the rates were 43% and 50%, respectively, for the patients with low MYL9 expression. We further examined the prognostic value of MYL9 expression in tumor cells in different subgroups of patients with ESCC stratified according to tumor differentiation, T classification, and lymph node metastasis. OS was significantly shorter in patients with high MYL9 expression in the moderate/well tumor differentiation subgroup (*n* = 99, *p* = 0.002; Fig 4C), T3+T4 subgroup (*n* = 103, *p* = 0.001; Fig 4D), and in patients without lymph node metastasis (*n* = 67, *p* = 0.009; Fig 4E). This was not the case for the poor tumor differentiation subgroup (*n* = 37, *p* = 0.507; S4A Fig), the T1+T2 subgroup (*n* = 33, *p* = 0.179; S4B Fig), and the lymph node metastasis subgroup (*n* = 69, *p* = 0.07; Fig 4F).

## Discussion

In this study, we reveal via IHC the frequent aberrant expression of MYL9 in ESCC tissues. This expression pattern was associated with ESCC histological differentiation. More importantly, we demonstrate that this MYL9 expression pattern in ESCC tissues was associated with poor prognosis and recurrence after curative resection. Furthermore, multivariate analyses revealed that elevated MYL9 expression in tumor cells was an independent and significant risk factor affecting recurrence and survival after curative resection.

Clinical evidence indicates that MYL9 expression is decreased in patients with bladder cancer, colon cancer, non–small cell lung cancer, and prostate cancer [17–20]. MYL9 downregulation is associated with unfavorable prognosis in patients with colon and prostate cancer [18, 20], which is opposite to our result Studies have shown the functional role of myosin II may differ from cancer to cancer. For example, MYH9, the heavy chain of myosin II, acts as a tumor suppressor in squamous cell carcinomas of the head and neck [18], while it promotes invasive behavior in breast tumor cells [9]. The biological role of MYL9 may be specific to disease. Besides, most of previous studies analyzed MYL9 expression using total mRNA or protein from human tissues. Here, we examined MYL9 expression based on its presence in the tumor cell cytoplasm without consideration of stromal portion expression. The different assessment methods may also contribute to the contradictory results. Even though it has been reported that MYL9 is present in the nuclei of human colonic circular smooth muscle cells and acts as a core transcription factor [22], we did not observe significant nuclear staining of MYL9 in these ESCC tissues.

The poor clinical outcome of patients with ESCC is attributed to the high rates of local invasion and regional lymph node metastasis [23]. MYL9 is involved in regulating breast cancer invasion [9, 16]. In MDA-MB-231 cells, MYL9 depletion did not affect cell cycle progression or induce cell death, but significantly reduced invasiveness [9]. In the present study, MYL9 expression levels were often higher in tumor cells at the invasion edge of the ESCC tissues (S2 Fig), suggesting that MYL9 may play a role in ESCC invasion. We speculate that MYL9 is a possible therapeutic target for preventing invasion in patients with ESCC with high MYL9 levels in the primary tumor cells.

Ser19 and/or Ter18 phosphorylation of MYL9 is crucial for its activation[24]. MYL9 activity, but not total levels of MYL9 protein, correlated with the invasive potential in breast cell lines [25]. Although treatment protocols that reduce MYL9 phosphorylation are beneficial for reducing the progression of invasion in hepatoma and ovarian cancer [26, 27], dephosphorylation of myosin regulatory light chain (MYL9) by Y27632 induced an invasive phenotype in low invasive lung adenocarcinoma cell lines [28]. Therefore, the effects of MYL9 phosphorylation appear to depend on the type of cancer cell. In fact, MYL9 phosphorylation levels in primary tumor tissues, including in ESCC, have not been addressed. Further studies are needed to investigate its role in the context of tumor progression.

High MYL9 expression is associated with lymphatic metastasis in non–small cell lung cancer[19]. However, we observed no correlation between MYL9 expression and regional lymph node metastasis. MYL9 expression levels were most closely related to tumor differentiation among the conventional clinicopathological characteristics of ESCC. The Rho/Rho-associated serine-threonine protein kinase (ROCK) pathway is involved in the differentiation of osteoblasts, lung fibroblasts, and other cell types [29, 30]. Inhibiting RhoA and ROCK activity partially restoreds osteogenic differentiation by inhibiting Hh-RhoA-Cofilin/MYL9 signaling [29]. Nevertheless, the effect of MYL9 on tumor cell differentiation has not been investigated. Additional research regarding MYL9 expression and status for tumor cell differentiation in ESCC is required.

In conclusion, MYL9 expression is significantly upregulated in ESCC tumor cells compared to adjacent non-tumor epithelial cells, especially in poorly differentiated tumors. Patients with high MYL9 expression in tumor cells have poor OS and recurrence-free survival. Our study suggests that MYL9 expression in tumor cells might be a promising prognostic biomarker and a potentially viable therapeutic target in ESCC.

## Supporting information

S1 Fig Representative staining of MYL9 and α-SMA in peritumoral regions of ESCC tissues. (TIF)

S2 Fig Representative images of MYL9 IHC staining at the edge of the tumor tissues. (TIF)

S3 Fig Representative images of MYL9 IHC staining in cervical, ovarian, colorectal, and hepatocellular carcinoma. (TIF)

S4 Fig Kaplan–Meier curves of patients (A) with poor tumor differentiation and (B) with T1+T2 classification.
(TIF)

## Abbreviations

MYL9: myosin light chain 9
ESCC: esophageal squamous cell carcinoma
ANT: adjacent noncancerous tissues
OS: overall survival
RFS: recurrence-free survival
IHC: immunohistochemistry
HR: hazard ratio
CI: confidence interval
UICC/AJCC: American Joint Caner Committee/Union International Contre le Cancer
DSMZ: Deutsche Sammlung von Mikroorganismen und Zellbulturen
DMEM: Dulbecco’s modified Eagle’s medium
FBS: fetal bovine serum
N.S.: not significant
CT: chemotherapy
RT: radiotherapy
CRT: combination of CT and RT (chemoradiotherapy)
