# Supplementary figures and images for: Expression and prognostic significance of MYL9 in esophageal squamous cell carcinoma
Source: PLoS One. 2017 Apr 7;12(4):e0175280. doi: 10.1371/journal.pone.0175280 (PMC5384754; doi:10.1371/journal.pone.0175280)

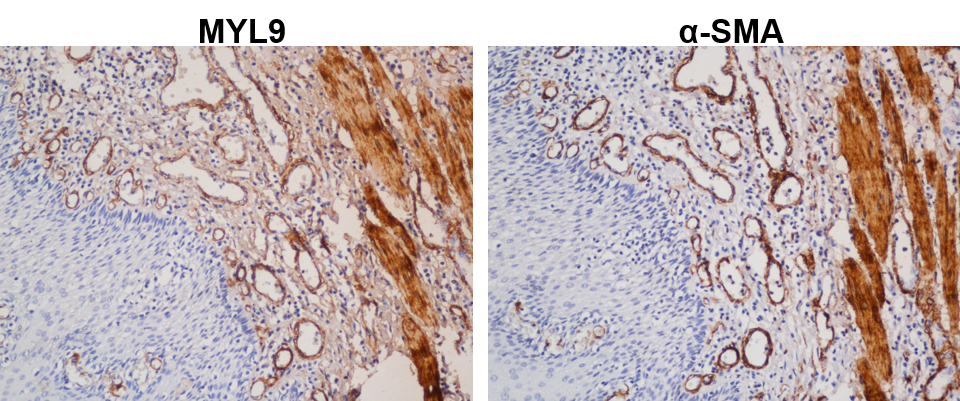

Supplement: S1 Fig — (TIF) [file pone.0175280.s001.tif]

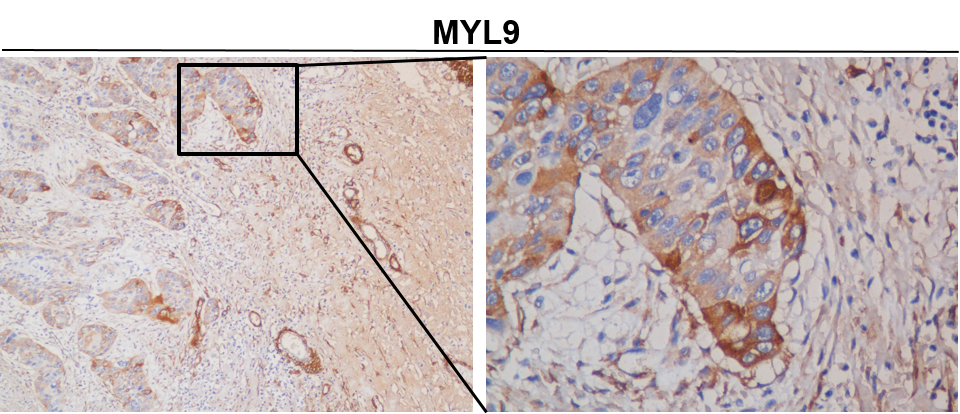

Supplement: S2 Fig — (TIF) [file pone.0175280.s002.tif]

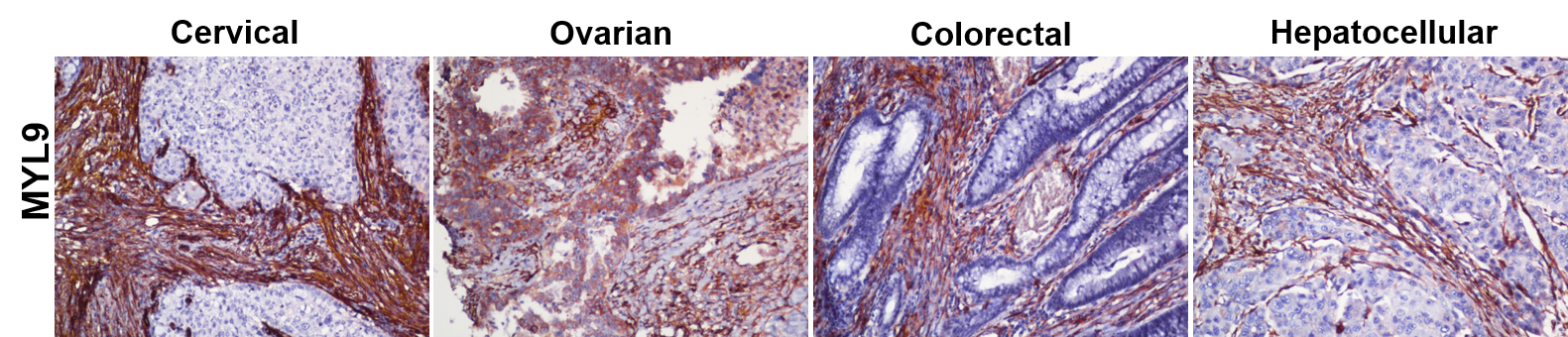

Supplement: S3 Fig — (TIF) [file pone.0175280.s003.tif]

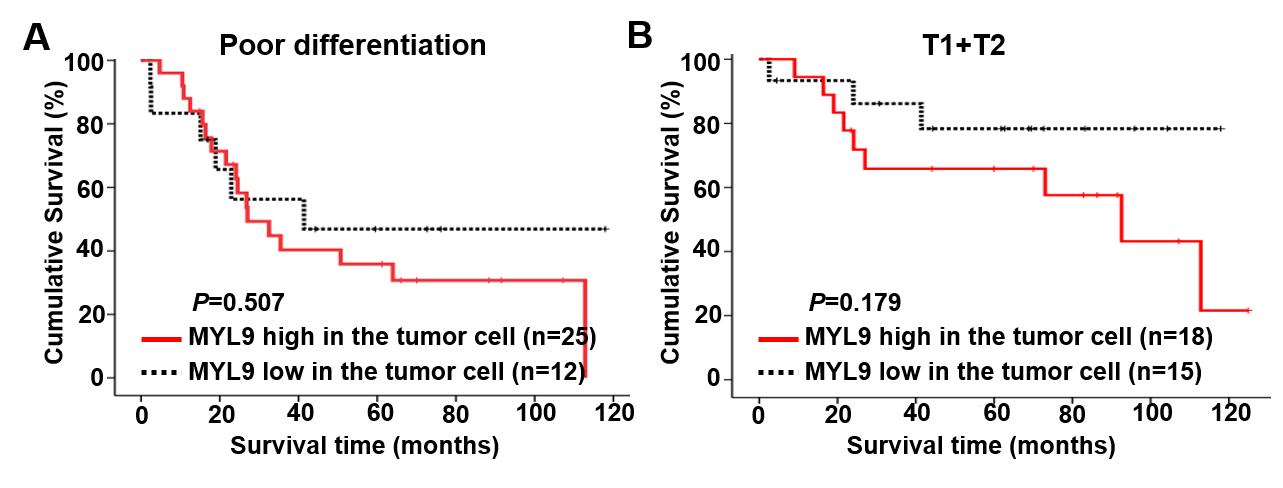

Supplement: S4 Fig — Kaplan–Meier curves of patients (A) with poor tumor differentiation and (B) with T1+T2 classification. (TIF) [file pone.0175280.s004.tif]
